# Factors Leading To Meconium Aspiration Syndrome in Term- and Post-term Neonates

**DOI:** 10.7759/cureus.5574

**Published:** 2019-09-05

**Authors:** Saroop Chand, Aamna Salman, Razia Mustafa Abbassi, Abdul Rehman Siyal, Fayaz Ahmed, Abdul Lateef Leghari, Ashmal Sami Kabani, Sajid Ali

**Affiliations:** 1 Pediatrics and Child Health, Aga Khan University Hospital, Karachi, PAK; 2 Obstetrics and Gynecology, Liaquat University Hospital, Hyderabad, PAK; 3 Pediatrics, Liaquat University Hospital, Hyderabad, PAK; 4 Pediatrics, Aga Khan University Hospital, Karachi, PAK

**Keywords:** meconium aspiration syndrome, fetal distress, intrauterine growth restriction, non-reassuring fetal heart rate

## Abstract

Background

Meconium aspiration syndrome (MAS) is considered a major cause of respiratory morbidity. It is a common issue encountered in the delivery room and newborn nursery. There is a need to identify the factors that lead to MAS to develop strategies to screen such patients at an early stage to decrease the mortality and morbidity. The objective of this study was to determine the factors leading to MAS in neonates delivered at ≥37 weeks of gestational age.

Methods

A cross-sectional study was conducted through non-probability consecutive sampling technique at Liaquat University Hospital, Hyderabad from August 2016 to February 2017. All neonates at ≥37 weeks of gestation with meconium-stained amniotic fluid (MSAF) detected during delivery were included in this study after obtaining informed consent from their parents. The demographic and factors related to MAS were recorded through predesigned proforma and analyzed using SPSS version 22. Mean and standard deviation were determined for quantitative variables whereas frequency and percentages were calculated for qualitative variables.

Results

Overall 136 neonates were included in the study. The mean gestational age was 38 ± 1.43 weeks. The major factors for MAS were detected as fetal distress (67.0%, n = 91), non-reassuring fetal heart rate (54.0%, n = 73), cesarean birth (48.0%, n = 65), intrauterine growth restriction (IUGR; 17.0%, n = 23), and post maturity (12.0%, n = 16).

Conclusion

We conclude that the major factors for MAS are fetal distress, non-reassuring FHR tracing, cesarean birth, IUGR, and post maturity. Screening of such patients at an early stage may minimize morbidity and mortality related to MAS.

## Introduction

Meconium aspiration syndrome (MAS) is an illness of the term and near-term newborn, which is associated with significant respiratory morbidity [1]. MAS is a common issue that most pediatricians experience in the delivery room and ordinary newborn nursery [1]. Passage of meconium-stained amniotic liquid (MSAF) during labor is estimated to occur at 12% to 15% in every live birth [2]. This figure is up to 24% in deliveries with maternal complications [3]. MAS occurs in around 5% of these pregnancies [2]. The figure has declined considerably from the earlier 22%, particularly in developed countries, accompanied by advances in obstetrics and neonatal care.

A study in an urban Pakistani population showed 27.3% of neonatal mortality, where there was a history or evidence of meconium passage during delivery [4]. It is as yet a noteworthy cause of mortality and morbidity in the developing nations [5]. With this in perspective Pakistan also has a high neonatal mortality rate (NMR) of 46 per 1000 live births [6]. The major risk factors for meconium-stained amniotic liquid (MSAF) and MAS include post maturity in 34% of cases, presence of fetal heart rate (FHR) irregularities in the intrapartum period in 51% of cases, cesarean birth in 42% cases, fetal distress in 77%, and intrauterine growth restriction (IUGR) in 6% of cases [1].

The rationale of the study that even after a robust search, no local data were found on factors leading to MAS and recent international data are scarce too; therefore, the present study was designed to generate local data. Hence, strategies could be devised to screen such patients at an early stage so that prompt intervention may halt or minimize morbidity and mortality related to MAS.

## Materials and methods

This cross-sectional study was conducted at the Liaquat University Hospital, Hyderabad from August 2016 to February 2017. A total of 136 neonates with gestational age equal to or greater than 37 weeks and who were born with meconium-stained liquor were included. Meconium-stained liquor is defined as the presence of meconium in amniotic fluid with a greenish or yellow discoloration of fluid. Inclusion criteria were a baby born to mother with a singleton pregnancy, gestational age equal to or greater than 37 weeks assessed on dating scan, and MSAF detected during delivery. Preterm babies with gestation <37 weeks, previous cesarean section, non-cephalic presentation, multiple pregnancies, congenital malformation, and intrauterine fetal demise were excluded. This study was conducted after the permission of the Ethical Committee of the hospital. Babies born with MSAF diagnosed during labor or delivery at the Liaquat University Hospital, Hyderabad were enrolled in this study. Prior to inclusion, informed consent for the study was obtained from the parents of eligible candidates. Data were collected on a predesigned proforma to assess the factors leading to MAS which included gestational age, mode of delivery, fetal distress, IUGR, non-reassuring CTG, gestational age on dating scan, fetal distress defined as APGAR score <7 at five minutes, and IUGR defined by fetal weight below the 10th percentile for gestational age after 24th week of gestation as determined through an ultrasound. Fetal heart tracing was evaluated, and non-reassuring fetal heart rate tracing was defined by either baseline bradycardia <110 or tachycardia >160 bpm, moderate to severe variable deceleration, late decelerations, loss of beat to beat variability, and prolonged fetal bradycardia. Finally, the child was labeled with MAS by defining MSAF in trachea assessed on suction and confirmed on chest X-rays showing overinflation of lungs with multiple opacities and flattening of the diaphragm.

After data collection, data were analyzed using Statistical Package for Social Science (SPSS) programming, Version 22. The mean and standard deviation were determined for quantitative factors like gestational age, APGAR score, and birth weight, and frequencies were calculated for mode of delivery (cesarean/vaginal delivery), presence of IUGR finding on last ultrasound, non-reassuring FHR, fetal distress, comorbidities of mother like history of hypertension, and diabetes mellitus. Impact modifiers such as gestational age and comorbidities such as infant of diabetic mother and hypertension were constrained by stratification and Chi-square test/Fischer exact test was conducted to understand the impact of these on result variables. *P* ≤0.05 was taken as significant.

## Results

A total of 136 neonates identified with MSAF were included in this study. Mean gestational age was 38 ±1.43, mean APGAR at five minutes and birth weight is also presented in (Table 1). Infants of a diabetic mother were 10 (7%) and 23 (17%) were born to mothers with a history of hypertension during pregnancy, as shown in Table 1.

**Table 1:** Demographics

	Mean (SD)	Frequency (%)
Gestational age (weeks)	38.01 (±1.43)	
APGAR score at 5 minutes	6.29 (±1.43)	
Birth weight (kg)	2.53 (±0.24)	
Infant of a diabetic mother		10 (7%)
Maternal hypertension		23 (17%)

In our cohort of 136 patients with meconium stained amniotic fluid, we found that 120 (88%) of the patients had a gestational age of 37-40 weeks, while 16 (12%) had a gestational age of > 40 weeks. (Table 2).

**Table 2:** Frequency and percentages of various risk factors leading to meconium aspiration syndrome FHR, fetal heart rate

Factors		Frequency (percentages)
Gestational age	>40 weeks	16 (12%)
	37-40 weeks	120 (88%)
Mode of delivery	Cesarean section	65 (48%)
	Vaginal delivery	71 (52%)
Fetal distress		91 (67%)
Intrauterine growth restriction		23 (17%)
Non-reassuring FHR tracing		73 (54%)

The major factors for MAS include non-reassuring FHR tracing found in 54% cases, cesarean birth in 48% cases, fetal distress in 67% of cases, IUGR in 16.9%, and post maturity (gestational age >40 weeks) in 11.8% of cases. The factors leading to MAS in neonates delivered at ≥37 weeks of gestation with respect to gestational age, maternal hypertension, and diabetes status are shown in Tables 3-5. 

**Table 3:** Association of gestational age with factors leading to meconium aspiration syndrome FHR, fetal heart rate

Risk Factors		Gestational age (weeks)		P-Value
		37 to 40	>40	
Mode of delivery	Cesarean section	49 (40.8%)	16 (100%)	<0.05
	Vaginal delivery	71 (59.2%)	0 (0%)	
Fetal distress		75 (62.5%)	16 (100%)	0.001
Intrauterine growth restriction		20 (16.7%)	3 (18.8%)	0.835
Non-reassuring FHR tracing		60 (50%)	13 (81.3%)	0.019

**Table 4:** Association of infant of diabetic mother with factors leading to meconium aspiration syndrome FHR, fetal heart rate

Factors		Infant of diabetic mother		P-Value
		Yes	No	
Gestational age >40 weeks		1 (10%)	15 (11.9%)	0.85
Mode of delivery	Cesarean section	3 (30%)	62 (49.2%)	0.33
	Vaginal delivery	7 (70%)	64 (50.8%)	
Fetal distress		6 (60%)	85 (67.5%)	0.73
Intrauterine growth restriction		1 (10%)	22 (17.5%)	0.54
Non-reassuring FHR tracing		3 (30%)	70 (55.6%)	0.119

**Table 5:** Association of maternal hypertension with factors leading to meconium aspiration syndrome FHR, fetal heart rate

Factors		Maternal Hypertension		P-Value
		Yes	No	
Gestational age >40 weeks		3 (13%)	13 (11.5%)	0.835
Mode of delivery	Cesarean section	9 (39.1%)	56 (49.6%)	0.361
	Vaginal delivery	14 (60.9%)	57 (50.4%)	
Fetal distress		17 (73.9%)	74 (65.5%)	0.434
Intrauterine growth restriction		4 (17.4%)	19 (16.8%)	0.946
Non-reassuring FHR tracing		13 (56.5%)	60 (53.1%)	0.764

## Discussion

The passage of MSAF during labor is reported to occur in 12% to 15% of all live births [4,7-8]. This figure is higher in deliveries associated with maternal complications (up to 24%) [3,9]. MAS occurs in around 5% of these pregnancies [2,9]. The figure has declined substantially from the earlier reported figure of 22%, especially in developed countries, accompanied by advances in obstetrics and neonatal care [8].

Pakistan’s NMR is 46 deaths per 1,000 live births [6]. According to a study in an urban Pakistani population, 27.3% of neonatal deaths had a history or evidence of meconium passage during delivery [9].

When exposed to stress such as hypoxia, fetus passes meconium and starts reflex gasping, a combination which allows meconium to reach the respiratory airways. The meconium then affects the respiratory system by mechanical airway obstruction, pneumonitis, and surfactant inactivation. All of these contribute to persistent pulmonary hypertension of the newborn (PPHN), which is the final common pathway for the severe morbidity and mortality seen in infants with MAS [3]. Meconium aspiration can present a diverse clinical spectrum ranging from mild self-resolving respiratory distress to severe respiratory failure resulting in severe morbidity and mortality. Severe meconium aspiration is also known to be associated with long-term abnormal respiratory reactivity [8]. The degree to which meconium has reached distal airways by the time of birth and the effect of meconium suction immediately after birth in preventing the severe clinical course are still controversial.

In the present study, major factors for MSAF-MAS include non-reassuring FHR tracing found in 53.7% cases, 47.8% cesarean birth, fetal distress in 66.9% of cases, IUGR in 16.9% cases, and post maturity (gestational age >40 weeks) in 11.8% of cases. Similar results were also reported in a study that stated major risk factors for MAS include post maturity in 34% of cases, presence of fetal heart rate abnormalities in the intrapartum period in 51% of cases, 42% in cesarean birth, fetal distress in 77% of cases and IUGR in 6% and maternal ethnicity (African American, Pacific Islanders) [1]. 

The relationship between the event of MSAF and fetal distress has been accounted for by many authors [10]. In a study by Yoder, infants with moderate to thick MSAF had substantially increased the occurrence of factors, suggestive of intrapartum compromise (irregular fetal heart pattern, fetal acidosis) in contrast with infants without MSAF and newborn children with light meconium staining of AF (*P *< 0.01) [11]. In a study by Berkus et al., the moderate and thick meconium groups had fundamentally higher danger of an unusual fetal heart rate tracing in each phase of labor and cord blood vessel pH under 7.20 (marker of fetal compromise) contrasting with the thin meconium and clear amniotic fluid (CAF) groups combined [12].

However, a study from Hyderabad, Pakistan refers to meconium being documented in 27.3% of neonatal deaths [4]. The study center was unable to offer proper mechanical ventilation to all the babies. Lack of ventilators combined with over-crowding, over-worked and inadequate staffing and inadequate or improper perinatal services are the main factors that need to be addressed [4,13-14]. Higher mortality in hospital-based studies usually mirrors the high-risk population, but comparative figures for developed countries are much lower.

## Conclusions

In this study, the major factors for MSAF and MAS include non-reassuring FHR tracing, cesarean birth, fetal distress, IUGR, and post maturity. MAS remains an important cause of morbidity as well as mortality in term and post-term newborns. In order to reduce the mortality and morbidity associated with MAS, strategies should be devised to screen such patients at an early stage so that prompt intervention may halt or minimize morbidity and mortality related to MAS.
